# Minimal Risk Doses of Cadmium Exposure Induce Histological and Functional Alterations in the Brown Adipose Tissue of Wistar Rats

**DOI:** 10.1007/s12011-025-04844-2

**Published:** 2025-10-01

**Authors:** Victor Enrique Sarmiento-Ortega, David Castillo-Pérez, Diana Moroni-González, Alfonso Diaz, Rubén Vázquez-Roque, Eduardo Brambila, Samuel Treviño

**Affiliations:** 1 Laboratory of Metabolomic and Chronic Degenerative Diseases, Physiology Institute, Meritorious Autonomous University of Puebla., Prol. de la 14 Sur 6301, Ciudad Universitaria, Puebla, C.P. 72560 Mexico; 2 Laboratory of Chemical-Clinical Investigations, Department of Clinical Chemistry, Chemistry Department, Meritorious Autonomous University of Puebla, 14 Sur. FCQ1, Ciudad Universitaria, Puebla, C.P. 72560 Mexico; 3 Laboratory of Neurochemistry and Behavior, Physiology Institute, Meritorious Autonomous University of Puebla., Prol. de la 14 Sur 6301, Ciudad Universitaria, Puebla, C.P. 72560 Mexico; 4 Laboratory of Neuroplasticity and Metabolism, Physiology Institute, Meritorious Autonomous University of Puebla., Ciudad Universitaria, Puebla, C.P. 72560 Mexico; 5 Faculty of Nursing, Meritorious Autonomous University of Puebla, Av. 25 Pte. 1304, Puebla, C.P. 72410 Mexico

**Keywords:** Cadmium toxicity, Brown adipose tissue, Thermogenesis, Mitochondrial complexes

## Abstract

Cadmium exposure, even at doses currently regarded as minimal risk, has been associated with significant metabolic alterations. While its effects on organs such as the liver, kidney, and pancreas have been widely studied, its impact on brown adipose tissue (BAT) remains poorly understood. In this study, we evaluated the effects of oral Cd exposure (15 and 32 ppm) over subacute, subchronic, and chronic periods on BAT functionality and structure in Wistar rats (n = 90). A metabolic, toxicological, and hormonal profile, as well as histology, expression of leptin, PPARγ, PPARα, and UCP-1, and mitochondrial complexes activity, were assessed and analyzed using one-way ANOVA and the Kruskal–Wallis test for quantitative and semiquantitative data. The results revealed progressive metabolic dysfunction, liver and renal impairment, increased free T3, and BAT dysfunction characterized by hypertrophy, downregulation of UCP-1 and PPARα, and disorganization of mitochondrial complexes and supercomplexes. These findings suggest a defect in BAT function and loss of its thermogenic capacity. In conclusion, results demonstrate that chronic Cd exposure induces mitochondrial toxic effects by impairing the function of brown adipose tissue and promoting the development of metabolic diseases, even under exposure levels considered to be of minimal risk.

**Supplementary Information:**

The online version contains supplementary material available at 10.1007/s12011-025-04844-2.

## Introduction

Cadmium (Cd) is an environmental pollutant without a physiological function in mammals and has a well-documented toxicological profile and carcinogenic effects [1]. In humans, exposure to Cd occurs primarily through the ingestion of contaminated food, inhalation of tobacco smoke, and contact with polluted water or air, especially in industrial areas or work environments where this metal is present. After entering the body, Cd tends to accumulate in tissues such as the liver and kidneys [2–5]. Chronic exposure, even at minimal risk levels (MRL), has been associated with a broad spectrum of adverse health outcomes, including metabolic disorders, insulin resistance, diabetes, and fatty liver [6–9]. Traditionally, Cd toxicological studies have focused on the acute effects of high doses. However, recent evidence suggests that prolonged exposure to MRL could affect tissues not considered classical sites of Cd accumulation. The MRL for Cd exposure is defined as an estimate of daily exposure to metal that is likely to be noncarcinogenic over a specified duration of exposure. Two MLR doses have been reported as the lowest observed adverse effect level (LOAEL) and the no-observed-adverse-effect level (NOAEL). Both levels depend on the exposure regarding duration time, concentration (dose), and the Cd toxicokinetics [6, 10–12].

Recently, we reported that adipose tissue is susceptible to the toxic effects of Cd at the minimal risk dose [11]. The body contains two main types of adipose tissue, each with distinct functions and characteristics. Histologically, white adipose tissue (WAT) is characterized by large, polygonal to spherical cells with a thin peripheral rim of cytoplasm, a flattened nucleus, and a large central lipid droplet that stores triglycerides. This tissue is formed by a meshwork of adipocytes, supported by a sparse extracellular matrix of collagen fibers and a network of capillaries. Their endocrine function is characterized by the synthesis and secretion of adipokines, which influence whole-body metabolism, inflammation, appetite, and other systemic processes. Clinically relevant adipokines include leptin and adiponectin, which regulate appetite, exhibit anti-inflammatory effects, and possess insulin-sensitizing properties [13]. However, when WAT is expanded in a dysfunctional manner, it can become pro-inflammatory, contributing to the development of insulin resistance, dyslipidemias, and other alterations that characterize metabolic syndrome [14].

On the other hand, brown adipose tissue (BAT) is a metabolically and thermogenically active tissue, densely vascularized and innervated to support its primary function of heat production through non-shivering thermogenesis. Histologically, BAT is composed of small and polygonal adipocytes with centrally located nuclei and a multivacuolated cytoplasm. Cytosolic features are attributed to BAT, which contains numerous small lipid droplets and a high abundance of mitochondria, resulting in a granular and acidophilic cytoplasmic appearance. The BAT mitochondria, together with peroxisome proliferator-activated receptor alpha and gamma (PPARα and PPARγ), play a crucial role in thermogenesis and energy expenditure. On the one hand, the activity of mitochondrial complexes and supercomplexes is associated with uncoupling protein 1 (UCP-1), a key protein that enables energy dissipation in the form of heat. Hormonal signals, such as thyroid hormones and catecholamines, finely control this process. Thyroid hormones (specifically the active form, triiodothyronine, called T3) directly stimulate the expression of UCP-1 [16].

On the other hand, PPARα is primarily involved in fatty acid oxidation, while PPARγ serves as a master regulator of adipogenesis, influencing both BAT development and glucose uptake and metabolism [15, 16]. BAT dysfunction has been linked to metabolic inflexibility, steatosis in multiple tissues, and increased cardiometabolic risk, as well as loss of BAT, commonly referred to as “whitening”. BAT whitening is common in obesity, characterized by the acquisition of unilocular features with triglyceride storage, which gradually lose their brown characteristics and assume WAT characteristics, thereby reducing substrate oxidation and leading to the loss of mitochondria through the impairment of molecular mechanisms regulating thermogenesis [17–19]. Nevertheless, the effects of low-dose Cd exposure on BAT have been studied little.

Given the emerging recognition of BAT as a critical metabolic organ, this study aimed to investigate the impact of oral Cd exposure at doses considered to be of minimal risk (15 and 32 ppm) in a time-dependent manner (subacute, subchronic, and chronic). We focused on evaluating BAT alterations in histomorphology, thermogenic protein expression, mitochondrial complex activity, and the organization of respiratory supercomplexes.

## Materials and Methods

### Animals and Treatment

A total of ninety male Wistar rats (70–80 g) were obtained from the “Claude Bernard” vivarium at the Universidad Autónoma de Puebla, Puebla. The animals were housed under controlled temperature conditions (22 °C) with a 12-h light/dark cycle and had unrestricted access to food and water. They were assigned to two groups and maintained on a standard caloric diet (5001, LabDiet; St. Louis, MO, USA) until they reached a body weight of 200 g. The control group (n = 30) had ad libitum access to Cd-free water, whereas the experimental groups (n = 60) were subdivided into two subgroups: one receiving drinking water containing 15 ppm of CdCl₂ (NOAEL dose) and the other 32 ppm of CdCl₂ (LOAEL dose), previously described [9, 11, 20–23]. All groups were kept under identical environmental and dietary conditions for durations of 15 days, 1, 2, 3, 4, and 5 months (n = 5 per subgroup). Whole blood samples were collected via cardiac puncture under anesthesia (xylazine/ketamine, 20/137 mg/kg) following a 4–5 h fasting period. Serum was obtained by centrifugation and stored at −70 °C for subsequent analysis. Interscapular BAT was excised, thoroughly perfused with cold saline, and stored at −70 °C, while a portion was fixed in 10% formalin for histological examination. All procedures were conducted following the Guide for the Care and Use of Laboratory Animals, established by the Mexican Council for Animal Care (NOM-062-ZOO-1999), and were approved by the Institutional Committee for the Care and Use of Animals. Compliance with both national and international standards ensured optimal animal welfare. Efforts were made to minimize the number of animals used and to reduce pain or discomfort throughout the study.

### Zoometry

At the end of each Cd exposure period, body weight, height, abdominal perimeter, body mass index (BMI), and visceral adiposity index (VAI) were measured. Body weight was measured using a digital scale (Torrey, Model: LPCR-20/40, Queretaro, Mexico). Abdominal perimeter was estimated using the distance between the diaphragm and the leg crease. BMI was calculated as follows: weight (g) divided by body size squared (cm), and VAI was calculated using a previously reported formula that incorporates abdominal perimeter, BMI, triglycerides, and high-density lipoprotein (HDL-C) concentration [24].

### Biochemical Assays in Serum

Serum levels of triiodothyronine (T3), thyroxine (T4), and insulin were quantified using commercial assay kits (SNIBE, Shenzhen, China) and read in an automated quimioluminescent MAGLUMI X3 (SNIBE, Shenzhen, China), according to the manufacturers’ protocols. Free fatty acids (FFA) levels were assessed following the method established by Brunk and Swanson [11]. Serum glucose, triglyceride, aspartate aminotransferase (ASAT), alanine aminotransferase (ALAT), and urea concentration were determined using commercial kits (Spinreact, Mexico City, Mexico) and an A25 autoanalyzer (BioSystems, Guadalajara, Mexico), employing standard spectrophotometric techniques. The homeostatic model assessment of insulin resistance (HOMA-IR) was determined as we previously reported [9].

### Adipose Tissue Enzyme-Linked ImmunoSorbent Assay

50 mg of the BAT was homogenized in ice-cold RIPA buffer with a protease inhibitor cocktail (Complete, Roche Applied Science). After homogenization with RIPA buffer, the samples were centrifuged at 4 °C at 3000 rpm for 10 min. The supernatant was used for non-nuclear proteins (leptin and UCP-1), and the pellet was resuspended in nuclear extraction buffer (ab113474) with constant shaking for nuclear proteins (PPARγ and PPARα) quantification. Finally, it was centrifuged at 15,000 rpm and 4 °C for 10 min, and the supernatant was separated. The concentrations of leptin (ab100773) and PPARγ (ab133101) were quantified using Abcam commercial kits (Abcam, Cambridge, MA). PPARα (MBS260518) was quantified using commercial kits from MyBioSource (San Diego, California, USA). UCP-1 (NBP2-82,465) concentration using Novus Biologicals (Colorado, USA) commercial kits. The protein concentration was determined using the Sedmak and Grossberg method, and the concentration of BAT markers was expressed in ng or pg per milligram of tissue.

### Adipose Tissue Histology

According to standard procedures, paraffin-embedded BAT tissue Sects. (9 μm thick) were deparaffinized and rehydrated. Hematoxylin and eosin (H&E) staining was performed to evaluate general tissue morphology. Photomicrographs were captured at 20X magnification using a light microscope (Leica Microsystems GmbH, Wetzlar, Germany). ImageJ software (National Institutes of Health, USA) was used to quantify relevant parameters, such as cell area and cell counts.

To perform immunofluorescence, tissue sections were deparaffinized and rehydrated. Afterward, antigenic recovery was conducted in Diva Decloaker 20X (Biocare Medical, CdMX, Mex) for 40 min at 60 °C. The samples were incubated with BSA (2%, Sigma-Aldrich; Toluca, Mex.) for blocking for 2 h at 4 °C. Subsequently, tissue sections were incubated overnight at 4 °C, with primary antibodies for Leptin (dilution 1:250; AB117751, Abcam, Cambridge, MA), PPAR-α (dilution 1:250; sc-398394, Santa Cruz Biotechnology Inc., CA, USA), PPAR-γ (dilution 1:250; sc-7273, Santa Cruz Biotechnology Inc., CA, USA), and UCP-1; (U6382 Sigma-Aldrich, Tol, Mexico). Leptin and UCP-1 were labeled with rhodamine as a secondary antibody (Jackson ImmunoResearch Laboratories, West Grove, PA, USA). FITC-conjugated secondary antibody (Jackson ImmunoResearch Laboratories, West Grove, PA, USA) was used for PPAR-α and PPAR-γ. Fluorescence microscopy, equipped with an integrated camera (Leica Microsystems GmbH, Wetzlar, Germany), was used to acquire images at a magnification of 20X. For semi-quantitative analysis, ImageJ software (National Institutes of Health, USA) was used, with the results expressed in arbitrary values.

### Activity of Mitochondrial Complexes

To reach mitochondrial isolation, brown adipose tissue was washed with cold PBS to remove residual blood. The tissue was transferred into a glass Elvehjem homogenizer containing Medium A (0.32 M sucrose, 10 mM Tris, 1 mM EDTA) and homogenized by up-and-down strokes using a motor-driven Teflon pestle. The homogenate was centrifuged at 1,000 × g for 5 min at 4 °C, and the supernatant was collected. It was centrifuged again at maximum speed for 10 min. The resulting pellet (BAT mitos) was resuspended in medium A, and protein concentration was determined using the Bradford assay. For the evaluation of mitochondrial complex activity, a pooled BAT sample from 5 animals per experimental group was used. A total of 200 µg of BAT mitos per group was resuspended in aminocaproic acid buffer and 10% digitonin (4 g/g of mitochondria). Samples were incubated on ice for 10 min and then centrifuged at maximum speed for 30 min at 4 °C. For blue native gel electrophoresis (BNGE), polyacrylamide gradient gels (3–13%) were prepared, and 100 μg of each sample was loaded into the wells of the gradient gel for enzymatic activity staining analysis. The gel was run at 90 V for 30 min, followed by 250 V for 90 min, until the dye front had migrated out of the gel. For in-gel activity assays, the following substrates were used: Complex IV (CIV), a 15 mL freshly prepared substrate containing 50 mg diaminobenzidine (DAB) and 100 mg cytochrome c in 50 mM phosphate buffer (pH 7.4), which was incubated for ~ 2 h. Complex I (CI): 20 mL of solution with 2 mM Tris–HCl, 0.1 mg/mL NADH, and 2.5 mg/mL nitro blue tetrazolium chloride (NTB), freshly prepared and incubated for ~ 1.5 h. Finally, gels were mounted between acetate sheets and scanned for densitometric analysis using ImageJ software.

### Statistical Analysis

The results are presented as the mean ± standard error of the mean (SEM). The Shapiro–Wilk test was applied to assess data normality. A two-way repeated measures ANOVA followed by Bonferroni's post hoc test was used to evaluate the effects of time on all parameters analyzed in exposed Cd groups (LOAEL and NOAEL doses). Their interaction was informed by the F-statistic value, calculated as the mean square of the time factor divided by the residual mean square, considering the degrees of freedom to determine the F-statistic distribution. All analyses were conducted using GraphPad Prism 8 (GraphPad Software Inc., USA). A significant level of p ≤ 0.05 was considered in comparison to the respective control group. Mitochondrial activity was not statistically analyzed.

## Results

### Effect of Cadmium Exposure on Zoometry, Biochemical, and Hormonal Parameters

**Fig. 1:**
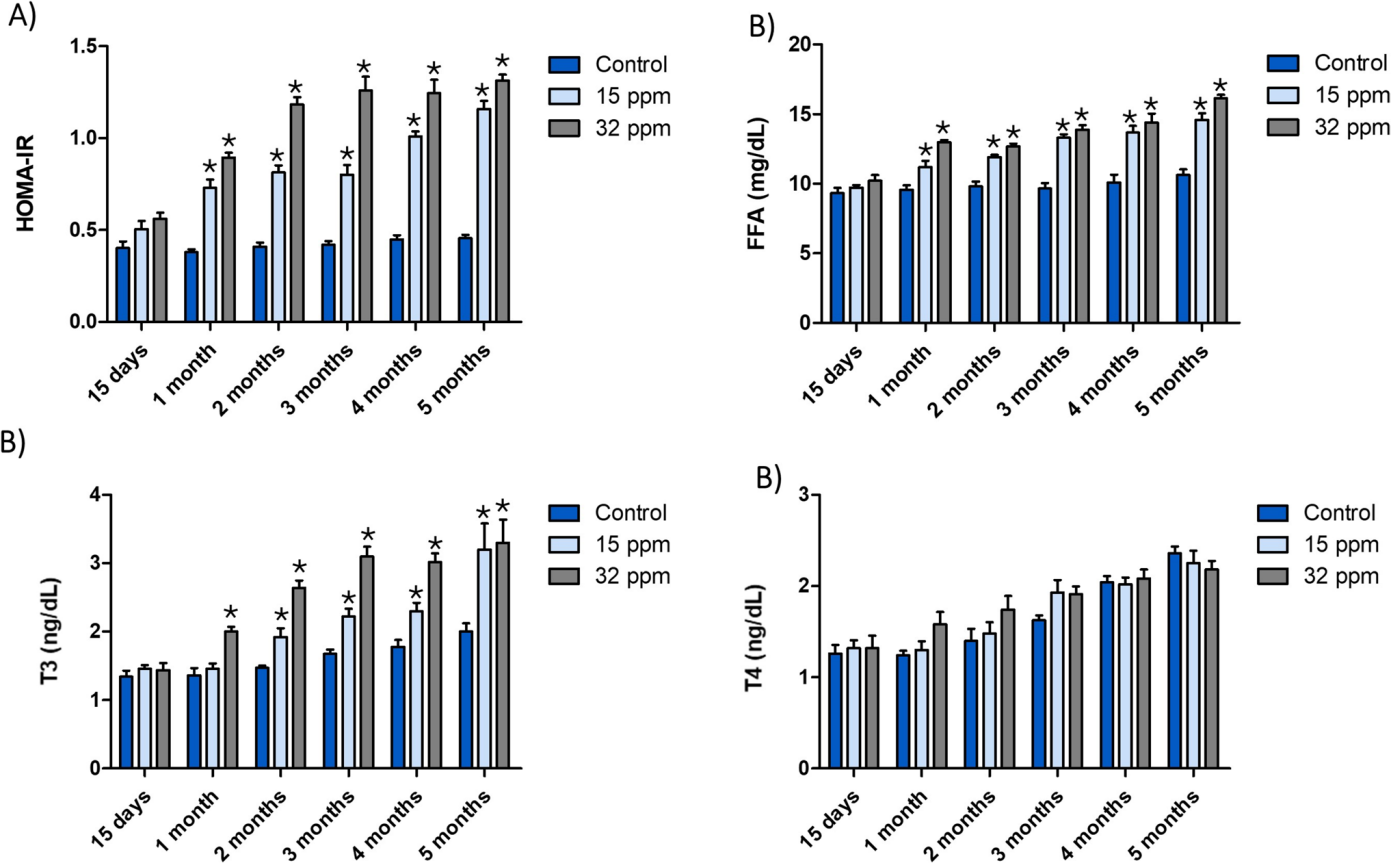
Effects of cadmium exposure on insulin resistance, circulating free fatty acids, and thyroid hormone profiles. **A**) The homeostatic model assessment of insulin resistance (HOMA-IR). **B**) Free fatty acids (FFA). **C**) Triiodothyronine (T3). D) Thyroxine (T4). The results shown are the average of 5 different experiments ± SEM. (*) indicates a significant difference regarding control groups (p ≤ 0.05) by a two-way repeated measures ANOVA followed by a Bonferroni post-test

Exposure to MRLs of cadmium from the third month induced an increase in body weight [F (10, 60) = 5.752; P < 0.0001] and abdominal perimeter [F (10, 60) = 13.15; P < 0.0001]. However, VAI increased from the second month of Cd exposure to MRLs [F (10, 60) = 7.664; P < 0.0001] (Table S1). Impairment of serum glucose [F (10, 60) = 1.672; P = 0.1087], triglyceride [F (10, 60) = 8.321; P < 0.0001], HDL-C [F (10, 60) = 1.897; P = 0.0633], and insulin concentration [F (10, 60) = 1.767; P = 0.00866] was observed from the first month of Cd exposure to both MRL doses. Hepatic enzymes (ASAT [F (10, 60) = 12.86; P < 0.0001] and ALAT [F (10, 60) = 2.744; P = 0.0075]) increased from the third month. Meanwhile, urea levels increased in both Cd-exposed groups from the fourth month [F (10, 60) = 2.813; P = 0.0063] (Table S2). Considering the relevance of systemic insulin resistance in metabolically active tissues, including brown adipose tissue, the HOMA-IR index was evaluated following cadmium exposure (Fig. 1A). From the first month onward, a progressive and dose-dependent increase in HOMA-IR [F (10, 60) = 7.192; P < 0.0001] and FFA [F (10, 60) = 5.560; P < 0.0001] levels was observed in both Cd exposure groups (Fig. 1B).

Since T3 is a key modulator of BAT thermogenesis and lipid metabolism, we quantified free triiodothyronine levels in Cd-exposed animals (Fig. 1C). Free T3 levels increased in a time- and dose-dependent manner in the 15 ppm group from 7 to 60%, while 32 ppm group increased 43% in the first month and reached 65% at five months to exposure [F (10, 60) = 3.966; P = 0.0003]. In contrast, the analysis of free thyroxine did not reveal statistically significant differences between Cd-exposed groups and controls at any time point (Fig. 1D).

### Cadmium Exposure Alters BAT Histomorphometry

**Fig. 2:**
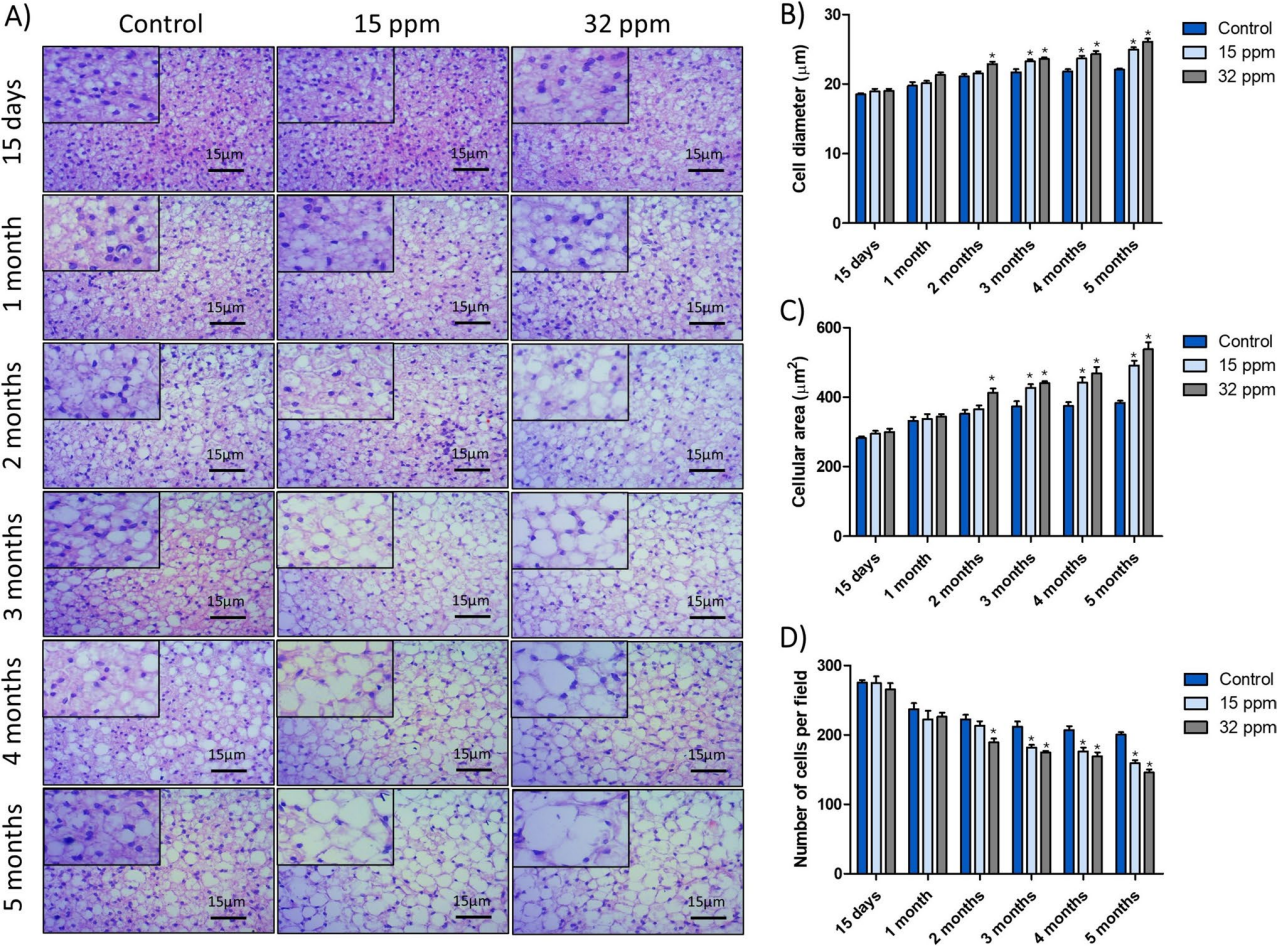
Effects of cadmium exposure on histomorphometry of brown adipose tissue. **A**) Hematoxylin–Eosin stain. **B**) Adipocyte diameter. **C**) Adipocyte area. **D**) Adipocyte number per field. The results shown are the average of 5 different experiments ± SEM. (*) indicates a significant difference regarding control groups (p ≤ 0.05) by a two-way repeated measures ANOVA followed by a Bonferroni post-test

To investigate the BAT morphology, the diameter of brown adipocyte cells was measured at each time point (Fig. 2B). Both MRLs induced a progressive increase in adipocyte size in a dose and time-dependent manner. Significant changes were observed from the third month of exposure in the 15 ppm group, with a 7.4% increase in cell diameter. Meanwhile, the 32 ppm group showed an increase in cell diameter of 9.2% to 18.1% [F (10, 375) = 10.90; P < 0.0001]. Similarly, cell area increased by 15% to 28% in the 15 ppm group and 18.0% to 40.2% in the 32 ppm group [F (10, 360) = 12.88; P < 0.0001] (Fig. 2C). BAT hypertrophy occurs at the chronicity of MRLs of Cd exposure. Also, it undergoes whitening, developing unilocular droplets that resemble WAT (Fig. 2A).

Additionally, we quantified the number of brown adipocytes per microscopic field, observing a progressive decline in cell number in both cadmium-treated groups compared to the controls (Fig. 2D). The decrement became more pronounced over time, and it was more evident in the higher-dose group. In the 15 ppm group, the decrease was 14.2% in the third month, and it remained at 20.6% after the fifth month. Meanwhile, in the 32 ppm group, the number of cells per field decreased by 15% from the second month to 27.2% in the fifth month [F (10, 360) = 8.898; P < 0.0001].

### Cadmium Exposure Impairs Leptin, PPARγ, PPARα, and UCP-1 Expression in BAT

**Fig. 3:**
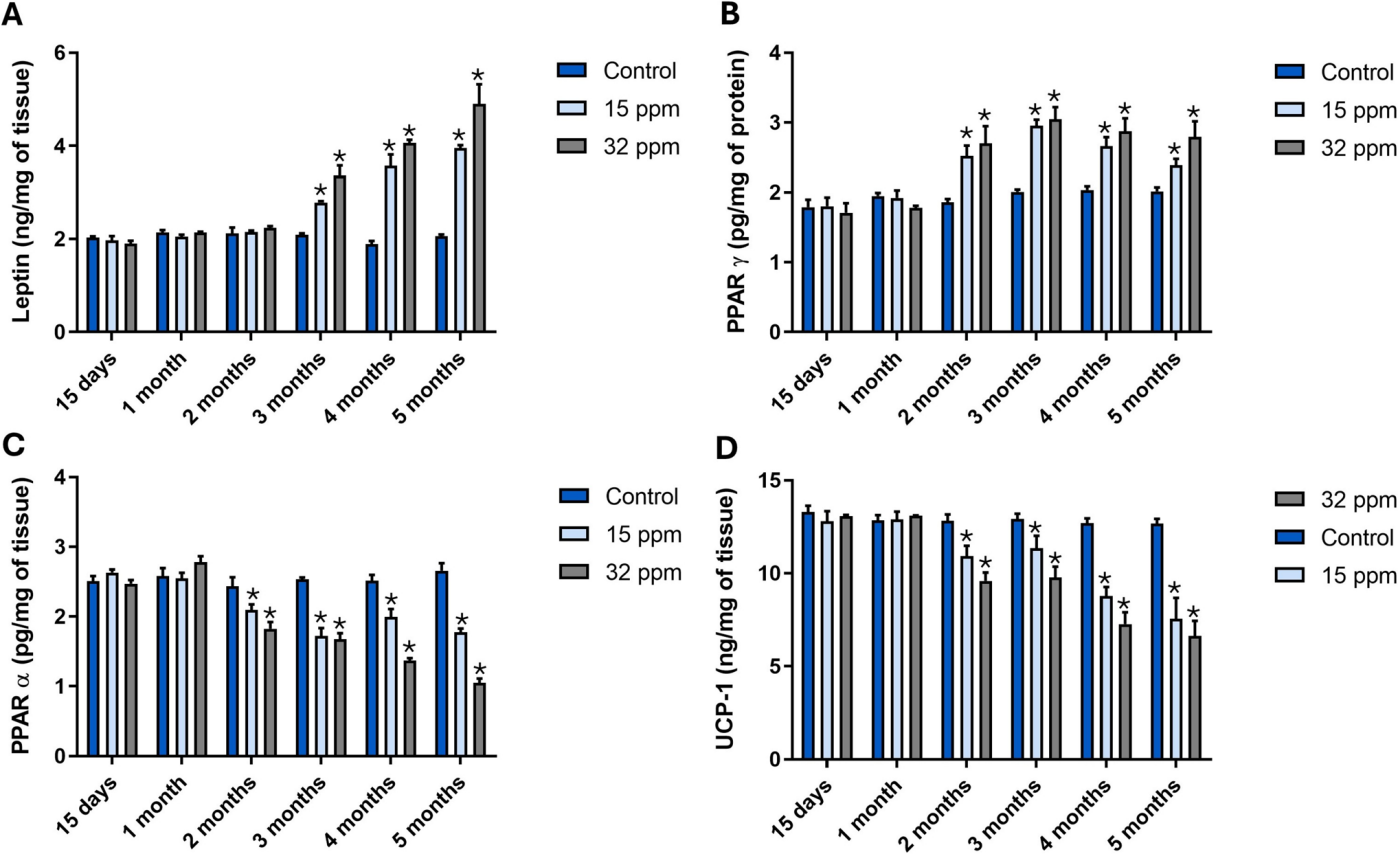
Leptin, PPARɣ, PPARα, and UCP-1 protein quantification in brown adipose tissue after cadmium exposure. **A**) Leptin. **B**) PPARɣ. **C**) PPARα. D) UCP-1. The results shown are the average of 5 different experiments ± SEM. (*) indicates a significant difference regarding control groups (p ≤ 0.05) by a two-way repeated measures ANOVA followed by a Bonferroni post-test

In the 15 ppm group, leptin increased progressively from 8.3% in the second month to 54.1% at the fifth month. Meanwhile, the 32 ppm group showed increases, from 41.6% in the second month to 91.6% at the fifth month [F (10, 30) = 23.56; P < 0.0001] (Fig. 3A). Leptin immunoreactivity in BAT increased from the third month in both Cd-exposed groups [F (10, 165) = 7.962; P < 0.0001] (Fig. S1). Likewise, a notable rise in PPARγ concentration was observed in both Cd-exposed groups by 18.2%—21.0% (15 ppm) and 20.6% to 43.5% (32 ppm; Fig. 3B) [F (10, 30) = 4.982; P = 0.0003]. In concordance, PPARγ immunoreactivity increased by 21.7% to 42.1% in both Cd-exposed groups, over time [F (10, 195) = 3.163; P = 0.0009] (Fig. S2).

Conversely, the PPARα protein quantification in the 15 ppm group diminished from 12.0% in the second month to 28.0% at the fifth month. In the 32 ppm group, the decreases were more pronounced by 24.0% in the second month to 56.0% in the fifth month [F (10, 30) = 17.55; P < 0.0001] (Fig. 3C). Meanwhile, PPARα immunoreactivity showed a pronounced downregulation from the second month in the 15 ppm group (16.7% to 30.2%), while in the 32 ppm group, it decreased by 34.6% to 54.8% [F (10, 240) = 4.077; P < 0.0001] (Fig. S3). The UCP-1 concentration decreased from 15.0% to 41.6% in the 15 ppm group and by 25.0% to 53.3% in the 32 ppm group [F (10, 30) = 6.792; P < 0.0001] (Fig. 3D). In concordance, the UCP-1 expression was progressively and dose-dependently suppressed in response to Cd exposure, with the suppression lasting up to 3 months (Fig. S4). From the second month, the 15 ppm and 32 ppm groups exhibited reductions in UCP-1 expression by 15.2% to 68.7% and 25.8% to 60.4%, respectively [F (10, 300) = 8.590; P < 0.0001].

### Cadmium Exposure Affects the Activity of Mitochondrial Complexes and Supercomplexes in BAT

**Fig. 4:**
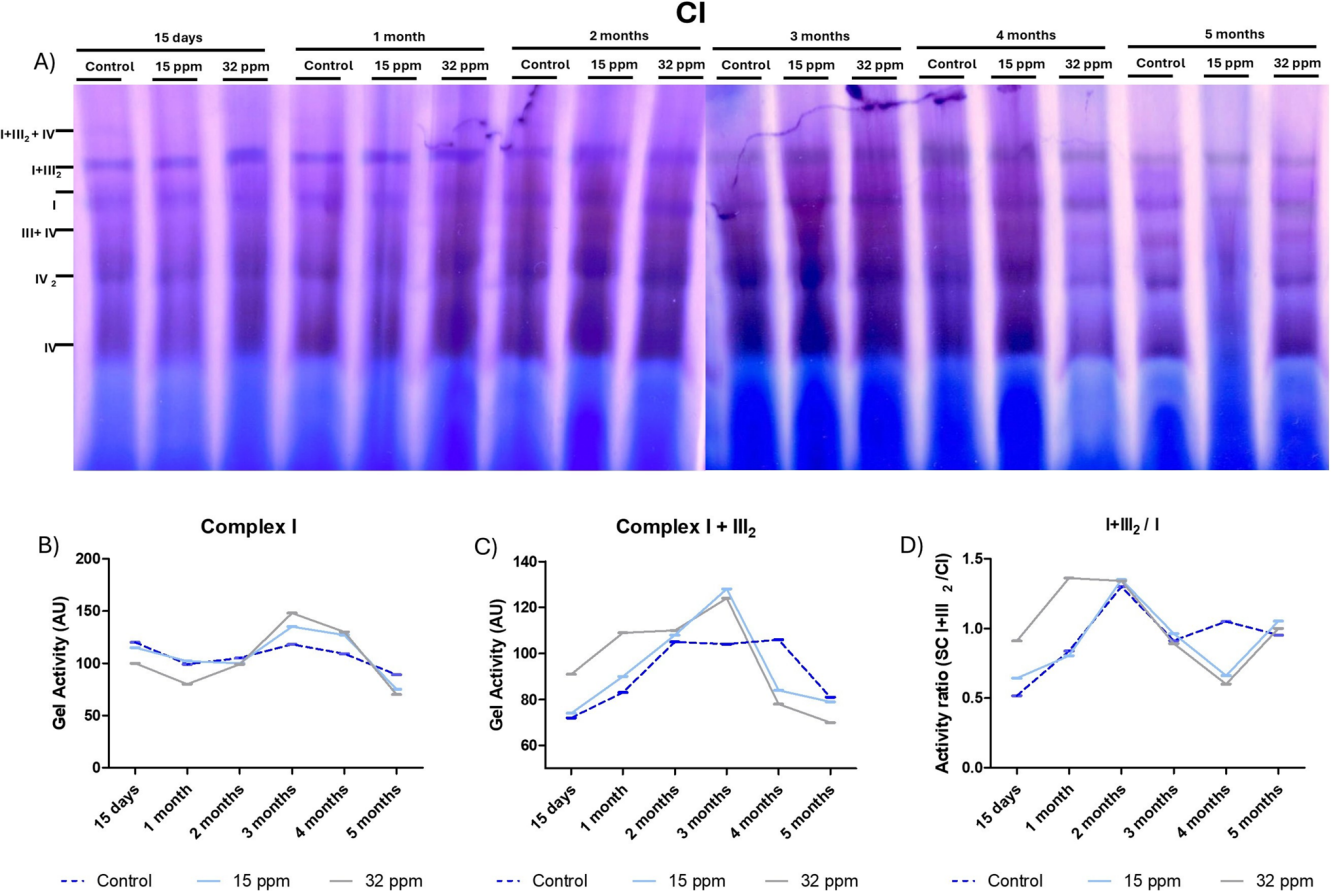
Mitochondrial Complex I activity of brown adipose tissue after cadmium exposure. **A**) BNGE showing the activity of CI (purple). **B**) Relative activity of complex I. **B**) Relative activity of complex I + III_2_. **D**) Relation of the activity of the super complex I + III_2_ and free I. The results show the analysis of a pooled sample (n = 5 per group)

We also evaluated mitochondrial functionality in BAT. Complex I activity was assessed over time (Fig. 4A). Cadmium exposure impaired enzymatic activity. At a 15-day time cohort, complex I activity was slightly reduced in both exposed groups. A slight increase occurred in the first and second months, particularly in the 32 ppm group, which continued into the third and fourth months. However, by the fifth month, activity dropped again (Fig. 4B). Meanwhile, the activity of the I + III₂ supercomplex increased markedly at early time points (15 days, mainly in the 32 ppm group). This augmentation was evident in both Cd-exposed groups until the third month. However, by the fourth and fifth months, supercomplex activity declined, falling below control levels in both Cd exposure groups (Fig. 4C). Additionally, we calculated the activity ratio of supercomplex I + III₂ to free complex I (I + III₂/I) (Fig. 4D). This ratio reflects the assembly efficiency and structural integrity of respiratory supercomplexes. At 15 days, both cadmium-exposed groups displayed an elevated ratio. A peak was observed at 1 month in the 32 ppm group, but not in the 15 ppm group. At 2 and 3 months, the I + III₂/I ratio was not different between groups. However, at the fourth month, the I + III₂/I ratio decreased again, and it recovered in the fifth month.

**Fig. 5:**
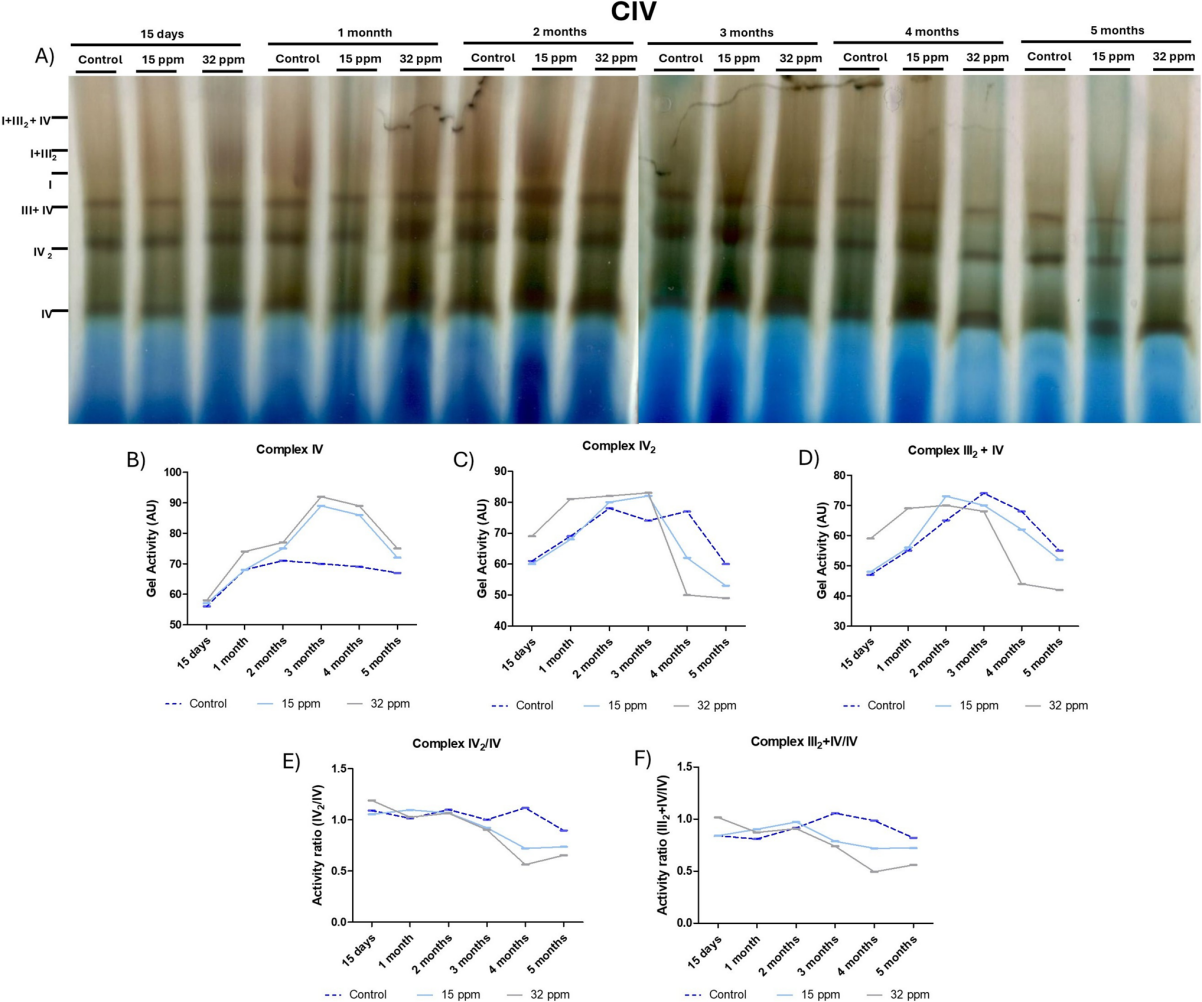
Mitochondrial Complex IV activity of brown adipose tissue after cadmium exposure. **A**) BNGE showing the activity of CI (purple). **B**) Relative activity of complex IV. **C**) Relative activity of complex dimer IV_2_. **D**) Relative activity of complex dimer III_2_ + IV. E) Relation of the activity of the super complex IV_2_ and monomer IV. F) Relation of the activity of the super complex III_2_ + IV_2_ and monomer IV. The results show the analysis of a pooled sample (n = 5 per group)

We also evaluated the complex IV activity and its related supercomplexes (Fig. 5A). The monomeric form activity of complex IV was increased in both Cd-exposed groups over time, with a peak at 3 months of analysis (Fig. 5B). The activity of the dimeric form of complex IV (IV₂) increased in the 32 ppm group in the first 3 months. Meanwhile, in the 15 ppm group, this augmentation was only observed from 2 to 3 months. Then, both Cd-exposed groups diminished the IV₂ complex activity (Fig. 5C). The activity of the mitochondrial supercomplex III_2_ + IV was increased during the first two months in both Cd-exposed groups. However, after the third month of Cd exposure to MRLs, the supercomplex III_2_ + IV low down activity was more evident in the 32 ppm group (Fig. 5D). To assess the structural organization of complex IV, we calculated the ratio of IV₂/IV, which was stable for the first 2 months, in both Cd-exposed groups; then activity diminished (Fig. 5E). Finally, to explore the integration of complex IV into higher-order assemblies, we calculated the ratio III₂ + IV/IV, which showed same behavior than IV₂/IV complex (Fig. 5F).

## Discussion

Our research group has previously demonstrated that chronic Cd-exposure to MRLs is associated with a wide range of adverse health effects, particularly the development of metabolic disorders [6, 9, 20–23, 25]. We have also shown that Cd-induced alterations in WAT architecture negatively impact key physiological functions, including oxidative stress regulation, inflammation, and abnormal adipokine secretion [9, 11]. Recently, transcriptomic dysregulation in BAT following chronic Cd exposure (100 ppm) has been reported, affecting its function and structure [10]. These effects are almost always associated with tissue damage resulting from Cd exposure to high concentrations. In contrast, our work focuses on evaluating the impact of oral Cd-exposure at MRLs (15 ppm and 32 ppm) under subacute, subchronic, and chronic conditions, and how these exposures influence specific functional and structural characteristics of BAT. According to our previous results in other tissues, there are compensation and adaptation mechanisms in function that do not demonstrate noticeable damage.

Zoometric and metabolic impairment were observed after exposure to MRLs of Cd (Table S1 and S2). A progressive increase in weight, abdominal perimeter, visceral adiposity index, serum glucose, triglycerides, FFA, insulin levels, and HOMA-IR indicated metabolic abnormalities induced by Cd, in a time- and dose-dependent manner (Fig. 1A and 1). Also, chronic toxic effects on the liver and kidney were observed in both Cd-exposed groups (Table S2). Together, these environmental conditions could affect BAT function due to an overabundance of nutrients and multi-tissue insulin resistance [7, 26].

Insulin resistance has been associated with significant BAT dysfunction, characterized by a reduction in its thermogenic capacity and glucose uptake. The insulin-resistant state limits the metabolic function of BAT and contributes to systemic energy imbalance. PET/CT studies using ^1^⁸F-FDG have shown reduced BAT activity in individuals with insulin resistance or type 2 diabetes, supporting a bidirectional relationship between BAT dysfunction and impaired insulin sensitivity [27, 28]. In rodents, BAT thermogenic activity relies on fatty acids for up to 90% of its energy production [29] [11]. Elevated serum FFA levels may indicate impaired lipid clearance or reduced thermogenic capacity of BAT under chronic Cd exposure conditions, regardless of the exposure dose.

The thermogenic capacity of BAT is classified into short- and long-term responses, depending on the duration and intensity of the stimulus. Thyroid hormones are potent inducers of BAT activity, acting both directly and synergistically with the sympathetic nervous system to enhance the transcription of thermogenic genes, including *UCP-1*, peroxisome proliferator-activated receptor γ coactivator 1α (*PGC-1α*), and type II iodothyronine deiodinase, which are essential for BAT differentiation and function [29]. Our results showed a persistent increase in free T3 (Fig. 1C), which may represent a compensatory mechanism aimed at enhancing energy expenditure. Notably, free T4 levels remained unchanged, suggesting that systemic thyroid hormone production is largely unaffected, and that observed alterations are likely due to peripheral regulation and enhanced T4 to T3 conversion in the liver despite transaminase augmentation. Interestingly, several reports have shown that Cd exposure, across varying doses and durations, leads to a reduction in thyroid hormone levels [30–32]. However, these findings contrast with our observations and thereby favour lipid droplet formation, which results in a BAT whitening.

In this way, we observed morphological changes in BAT (Fig. 2), which potentially reflect a shift toward a white-like adipose and a lipid-storing phenotype. An increase in brown adipose cell area and leptin expression suggests a whitening process or possible WAT-like transdifferentiation (Fig. 3A and S1). The BAT whitening process has significant metabolic and health implications that have received little attention. Emerging evidence suggests that BAT whitening is a multifactorial phenomenon influenced by various risk factors, including a poor diet, aging, and genetic background [19]. These factors not only promote weight gain but also impair BAT function. Although the underlying mechanisms remain incompletely understood, we propose that Cd exposure also compromises BAT function, with this effect being more pronounced after two months of exposure, regardless of the Cd dose.

Moreover, PPARγ and PPARα mediate the balance between burning and whitening of BAT. Both are key transcription factors that regulate the BAT function. Our results showed a fluctuation in PPARγ immunoreactivity (Fig. 3B, 3, S2, and S3), suggesting a dynamic regulatory response in BAT under Cd exposure. The mid-to-late-stage elevations may reflect a compensatory attempt to preserve adipocyte identity or promote lipid storage capacity in the context of hypertrophy and increased brown adipose cell area. It has been reported that PPARγ plays a crucial role in the differentiation and maintenance of brown adipocytes, supporting lipid storage [33]. In contrast, Zhu et al. found no changes in the expression of PPARγ mRNA in BAT; however, an increase in the expression of PPARα was observed after exposure to 100 ppm of Cd [10]. Probably, the inconsistencies between studies are due to variations in dose, exposure duration, species, and experimental design, which limit the ability to draw a conclusive interpretation. Additionally, our results showed a progressive loss of PPARα expression (Fig. 3C and S3), which is essential for promoting mitochondrial biogenesis and fatty acid oxidation. PPARα acts synergistically with thermogenic genes such as *UCP-1*, *PGC-1α*, and *Cidea*, coordinating the energy-dissipating activity of BAT [33, 34]. These dynamic changes indicate compromised oxidative capacity in BAT due to Cd exposure, which may contribute to the observed hypertrophy and reduced cellularity. They may reflect impaired mitochondrial function and fatty acid turnover in the context of chronic Cd toxicity.

The loss of PPARα expression in BAT after exposure to MRL of Cd could disrupt the thermogenic capacity of the tissue, explaining part of the probable mechanism by which Cd may impair energy homeostasis and cause metabolic disturbances. The downregulation of PPARα, in consequence, declines UCP-1 expression (Fig. 3D and S4). UCP-1 is the BAT hallmark protein. It is essential for thermogenesis, as it uncouples mitochondrial respiration from ATP production, allowing energy to be dissipated as heat. The reduction in UCP-1 expression by Cd exposure may result from oxidative stress, impaired T3 and β-adrenergic signaling, and metabolic disturbances [35–37].

Since thermogenesis is highly dependent on mitochondrial integrity, any alteration in mitochondrial complexes can compromise the energy efficiency of BAT, as observed in Cd-exposure groups. In this context, we measured the activity of complex I over time (Fig. 4). Cadmium exposure induced dynamic changes in complex I activity, suggesting an initial mitochondrial adaptation—possibly linked to UCP-1 downregulation—followed by a functional decline of BAT. A decrease in mitochondrial complex I activity reduces the efficiency of the electron transport chain, thereby limiting ATP production and increasing the generation of reactive oxygen species (ROS) [38]. Complementary, the supercomplex I + III₂ is a supramolecular structure that enhances electron transfer efficiency and minimizes ROS production during oxidative phosphorylation [39]. Our results showed that temporal changes in supercomplex I + III₂ activity mirror a biphasic pattern observed in the complex I, indicating early mitochondrial adaptation through improved respiratory coupling, followed by structural or functional deterioration during prolonged Cd exposure.

The efficiency of supercomplexes formation is analyzed by the activity ratio of supercomplex I + III₂ to free complex I (I + III₂/I). This ratio reflects the assembly dynamics and structural integrity of the respiratory supercomplex. Our results indicated an initial formation of the supercomplex, followed by destabilization, which supports the concept of time-dependent mitochondrial remodeling and subsequent decline in functional capacity under chronic stress induced by Cd. Additionally, the terminal oxidative capacity of the electron transport chain is responsible for the activity of monomeric complex IV, which catalyzes the final transfer of electrons to molecular oxygen, thereby enhancing respiratory efficiency [40]. Cadmium exposure induced a moderate but time-dependent increase in complex IV monomer activity, particularly at long times (Fig. 5). This finding suggests a potential compensatory upregulation of complex IV to maintain respiratory flux in response to upstream inefficiencies or altered supercomplex organization [39–41].

Cadmium exposure also elicited a biphasic response in complex IV dimer activity, characterized by an initial upregulation that potentially aims to support enhanced respiratory capacity. However, with prolonged exposure, the complex IV₂ activity declined, suggesting disruption of dimer assembly and stability, contributing to mitochondrial dysfunction at later stages. This pattern is consistent with a decrease in complex I activity and the disorganization of respiratory supercomplexes. Additionally, the activity of the mitochondrial supercomplex III₂ + IV is a hallmark of respiratory chain organization integrity and electron flux at the terminal stage of oxidative phosphorylation, observed as a biphasic pattern, initially augmented, followed by a decline, likely reflecting an early adaptive response to preserve electron transport efficiency. These adaptations are parallel to the changes observed in the activity of supercomplex III₂ + IV and the monomeric complex IV (III₂ + IV/IV), which is essential for efficient electron transfer and may be upregulated in response to increased energy demands. In BAT, it may lead to reduced thermogenic capacity and loss of proton uncoupling [41–43].

Although there is ample evidence on the chemical, biochemical, and molecular mechanisms by which Cd inhibits the activity of individual electron transport chain complexes in various tissues, such as the liver, kidney, and brain, its effect on the organization and functionality of mitochondrial supercomplexes in BAT has not yet been adequately explored. Therefore, our work could be the first to evaluate these effects, as supercomplexes not only optimize the efficiency of electronic transport to have a greater thermogenic effect but also reduce ROS generation. Thus, its alteration in BAT could have significant metabolic implications, particularly in processes such as thermogenesis and systemic energy balance.

However, our work has limitations; we did not perform a thermogenic capacity inspection to verify the BAT functional decline and the mitochondrial activity assay in each rat, as Cd exposure promoted BAT whitening, which significantly reduced the quantity of tissue available for analysis. As a result, we found ourselves in need of generating these assays from a sample pool per group in each time cohort. Additionally, the number of experimental subjects per group could lead to a high risk of a Type II error (false negative). However, the rationale for the n = 5 is sound for this experimental design because the study's goal was to chart the dose- and time-dependent effects of Cd on BAT. Finally, we performed this study only in male Wistar rats because sex hormone variations are more controlled in males than in females, where Cd acts as a sexual hormone disruptor. Despite this, we found interesting evidence to support new works in this field that elucidate the mechanisms involved.

In summary, this study demonstrates that oral Cd exposure, even at the MRL, consistently induces metabolic disturbances, toxic effects in the chronic phase, and an increase in free T3 levels, as well as progressive histomorphologic changes in BAT, likely to WAT, which are time- and dose-dependent. Additionally, a loss in the expression of key thermogenic factors, such as UCP-1 and PPARα. A disruption in the functional organization of mitochondrial complexes accompanies an increase in leptin and PPARγ expression. In conclusion, these results demonstrate that chronic Cd exposure exerts toxic effects by impairing the function of brown adipose tissue and potentially inducing the whitening process.

## Supplementary Information

Below is the link to the electronic supplementary material.

ESM 1 (JPG 656 KB)

ESM 2 (JPG 0.98 MB)

ESM 3 (JPG 1.23 MB)

ESM 4 (JPG 907 KB)

ESM 5 (DOCX 19.2 KB)

ESM 6 (DOCX 20.4 KB)

## Data Availability

No datasets were generated or analysed during the current study.
