# Supplementary figures and images for: Minimal Risk Doses of Cadmium Exposure Induce Histological and Functional Alterations in the Brown Adipose Tissue of Wistar Rats
Source: Biol Trace Elem Res. 2025 Oct 1;204(4):2631–42. doi: 10.1007/s12011-025-04844-2 (PMC13128740; doi:10.1007/s12011-025-04844-2)

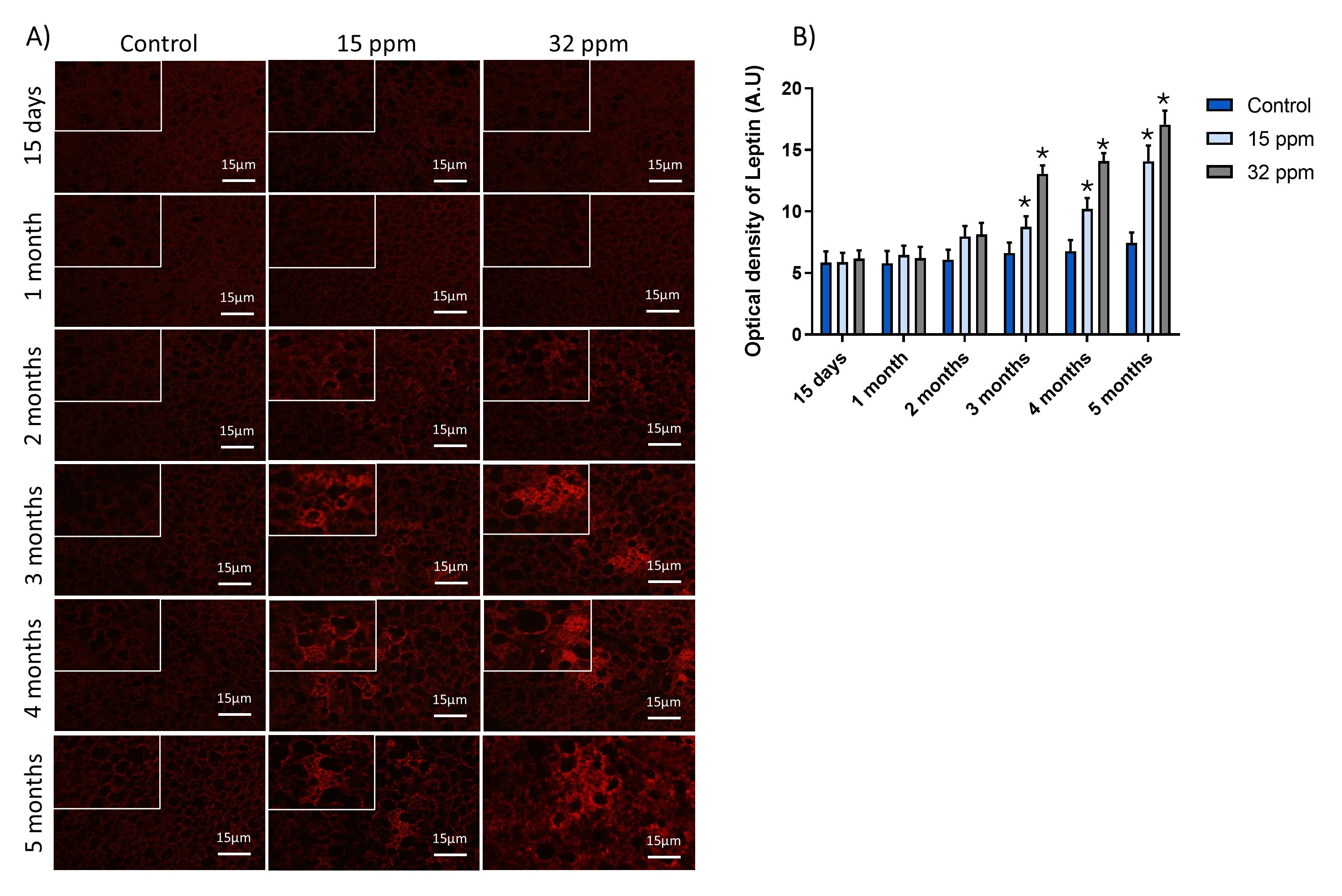

Supplement: Supplementary file 1 — (JPG 656 KB) [file 12011_2025_4844_MOESM1_ESM.jpg]

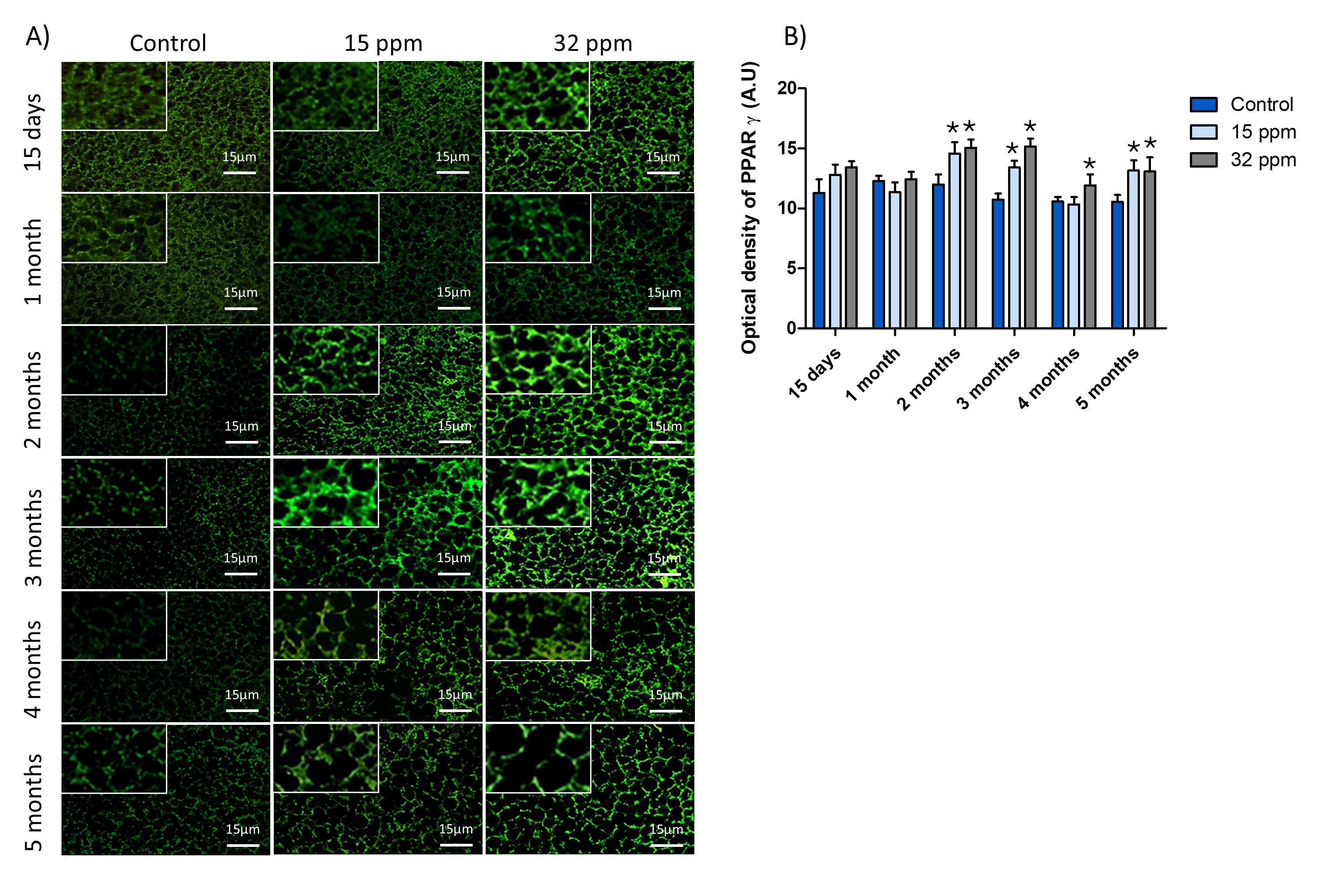

Supplement: Supplementary file 2 — (JPG 0.98 MB) [file 12011_2025_4844_MOESM2_ESM.jpg]

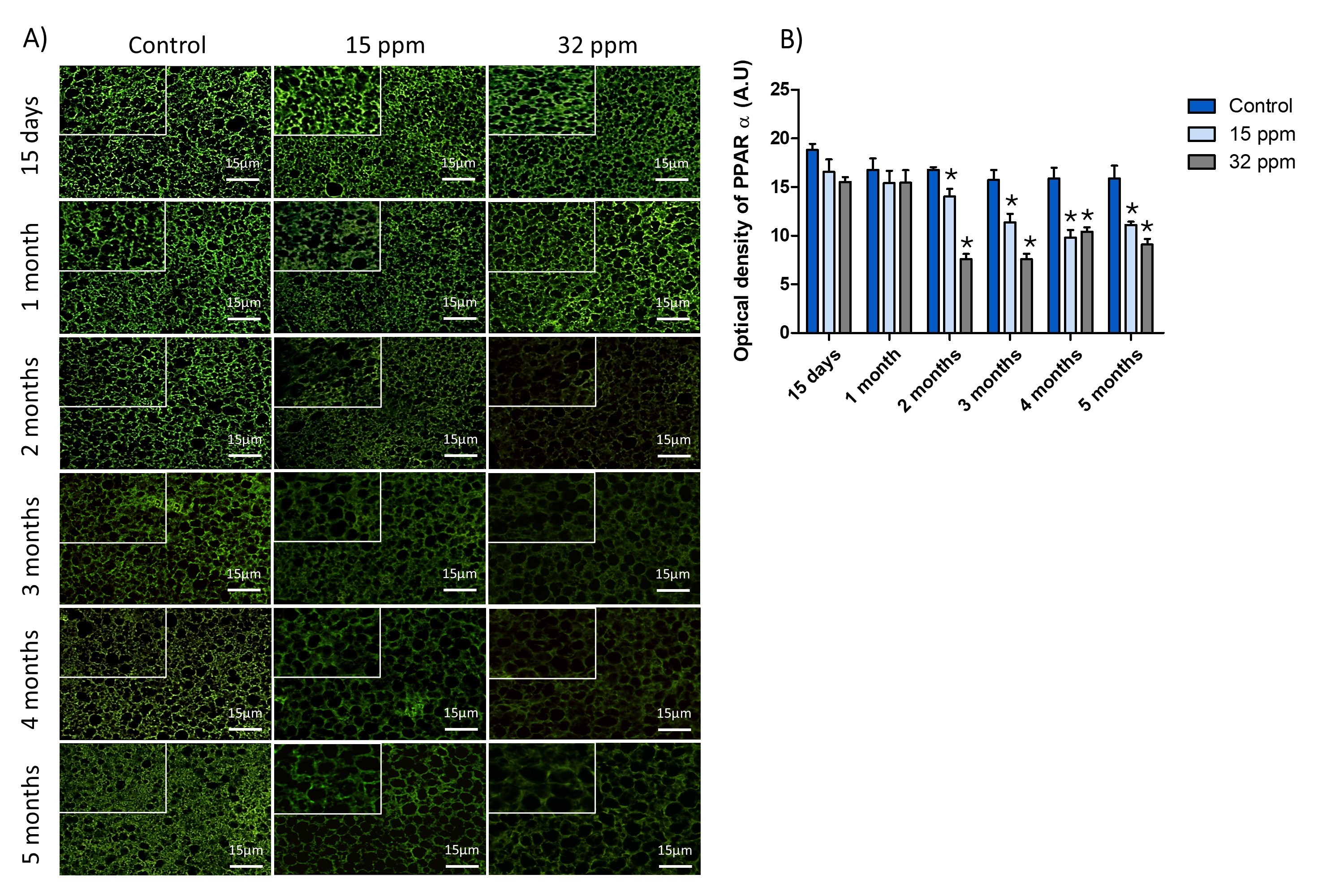

Supplement: Supplementary file 3 — (JPG 1.23 MB) [file 12011_2025_4844_MOESM3_ESM.jpg]

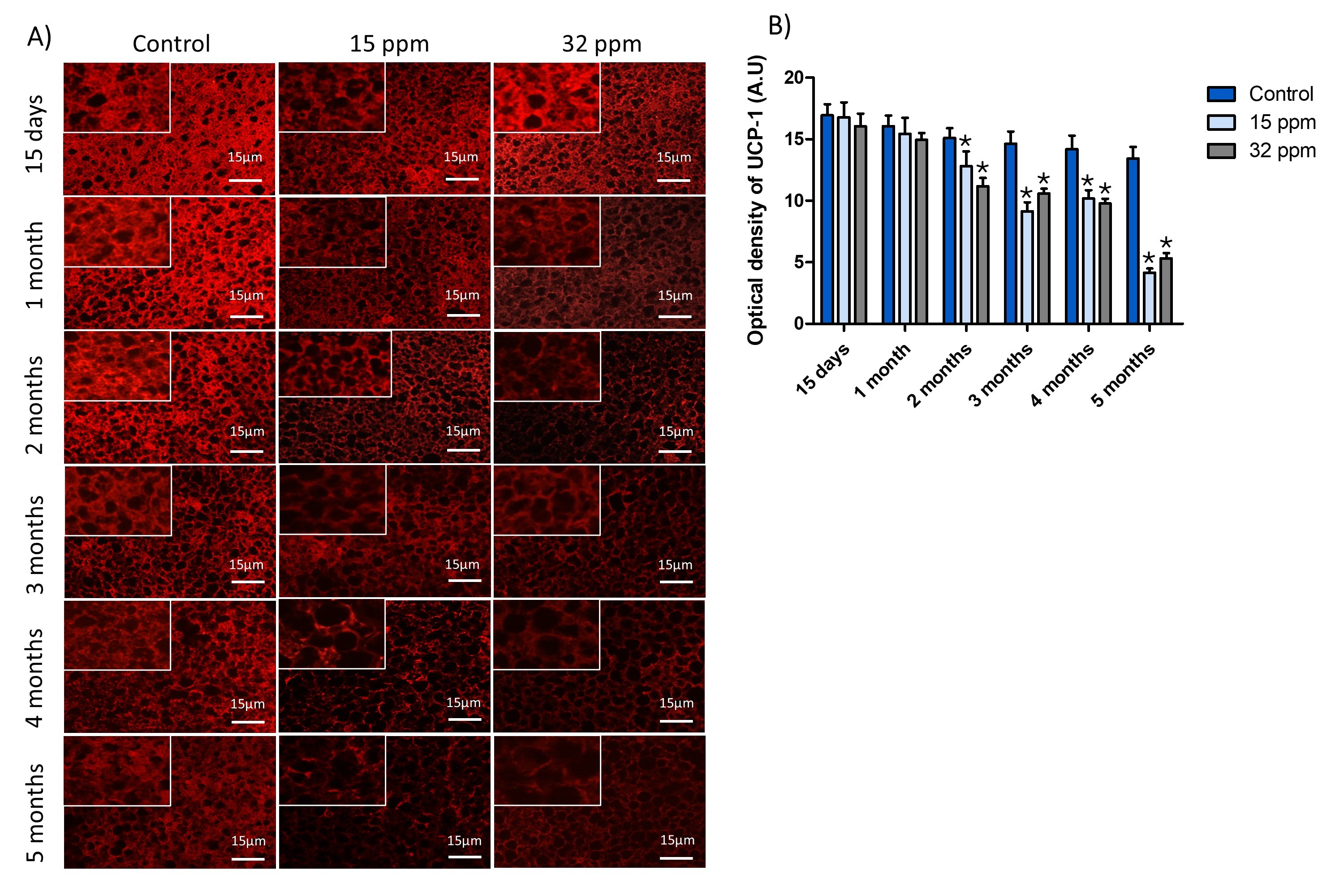

Supplement: Supplementary file 4 — (JPG 907 KB) [file 12011_2025_4844_MOESM4_ESM.jpg]
