# Supplementary material for: Minimal Risk Doses of Cadmium Exposure Induce Histological and Functional Alterations in the Brown Adipose Tissue of Wistar Rats
Source: Biol Trace Elem Res. 2025 Oct 1;204(4):2631–42. doi: 10.1007/s12011-025-04844-2 (PMC13128740; doi:10.1007/s12011-025-04844-2)
Supplement: Supplementary file 5 — (DOCX 19.2 KB) [file 12011_2025_4844_MOESM5_ESM.docx]

Table S1. Effect of Cadmium Exposure on Zoometric Parameters.

|  |  | Weight (g) | Height (cm) | Abdominal perimeter (cm) | Body mass index (g/cm^2^) | Visceral adiposity index |
| --- | --- | --- | --- | --- | --- | --- |
| 15 days | Control | 275 ± 9.3 | 15.6 ± 0.2 | 14.8 ± 0.2 | 1.2 ± 0.05 | 2.1 ± 0.1 |
|  | 15 ppm | 209 ± 6.9▼ | 15.1 ± 0.3 | 14.7 ± 0.4 | 0.8 ± 0.02▼ | 2.1 ± 0.09 |
|  | 32 ppm | 212 ± 4.5▼ | 15.2 ± 0.2 | 14.5 ± 0.3 | 0.9 ± 0.03▼ | 2.2 ± 0.2 |
| 1 month | Control | 313 ± 5.6 | 16.0 ± 0.5 | 14.9 ± 0.3 | 1.2 ± 0.03 | 2.3 ± 0.1 |
|  | 15 ppm | 311 ± 7.1 | 15.8 ± 0.3 | 15.1 ± 0.6 | 1.1 ± 0.02 | 3.5 ± 0.2▲ |
|  | 32 ppm | 304 ± 8.3 | 14.9 ± 0.6 | 14.8 ± 0.3 | 1.1 ± 0.04 | 4.4 ± 0.4▲ |
| 2 months | Control | 349 ± 5.2 | 17.1 ± 0.5 | 15.7 ± 0.2 | 1.23 ± 0.1 | 1.9 ± 0.1 |
|  | 15 ppm | 362 ± 7.3 | 17.3 ± 0.2 | 16.1 ± 0.3 | 1.1 ± 0.9 | 2.7 ± 0.1▲ |
|  | 32 ppm | 359 ± 6.7 | 16.8 ± 0.6 | 16.0 ± 0.5 | 1.1 ± 0.1 | 4 ± 0.3▲ |
| 3 months | Control | 375 ± 9.5 | 18.6 ± 0.5 | 16.4 ± 0.5 | 1.25 ± 0.08 | 1.8 ± 0.1 |
|  | 15 ppm | 401 ± 8.3▲ | 17.9 ± 0.4 | 18.6 ± 0.6▲ | 1.4 ± 0.1 | 3.9 ± 0.3▲ |
|  | 32 ppm | 408 ± 11▲ | 18.2 ± 0.3 | 19.1 ± 0.4▲ | 1.5 ± 0.1 | 4.7 ± 0.3▲ |
| 4 months | Control | 386 ± 8.5 | 19.9 ± 0.6 | 17.0 ± 0.6 | 1.25 ± 0.1 | 1.9 ± 0.1 |
|  | 15 ppm | 426 ± 11.3▲ | 19.0 ± 0.5 | 21.5 ± 0.5▲ | 1.3 ± 0.09 | 4.6 ± 0.2▲ |
|  | 32 ppm | 422 ± 12.4▲ | 20.2 ± 0.4 | 22.7 ± 0.4▲ | 1.25 ± 0.08 | 4.2 ± 0.5▲ |
| 5 months | Control | 415 ± 10.9 | 20.8 ± 0.4 | 18.1 ± 0.1 | 1.1 ± 0.1 | 2.8 ± 0.6 |
|  | 15 ppm | 452 ± 11.4▲ | 19.9 ± 0.8 | 22.8 ± 0.2▲ | 1.3 ± 0.1 | 6 ± 0.4▲ |
|  | 32 ppm | 448 ± 12.2▲ | 21.1 ± 0.7 | 23.4 ± 0.4▲ | 1.34 ± 0.09 | 5 ± 0.4▲ |

The results shown are the average of 5 different experiments ± SEM. The results shown are the average of 5 different experiments ± SEM. (▲/▼) indicates a significant difference between the control group and Cd-exposed groups by a two-way repeated measures ANOVA followed by a Bonferroni test. A p-value of ≤ 0.05 was considered statistically significant.
