# Supplementary material for: Minimal Risk Doses of Cadmium Exposure Induce Histological and Functional Alterations in the Brown Adipose Tissue of Wistar Rats
Source: Biol Trace Elem Res. 2025 Oct 1;204(4):2631–42. doi: 10.1007/s12011-025-04844-2 (PMC13128740; doi:10.1007/s12011-025-04844-2)
Supplement: Supplementary file 6 — (DOCX 20.4 KB) [file 12011_2025_4844_MOESM6_ESM.docx]

Table S2. Effect of Cadmium Exposure on Biochemical Parameters.

|  |  | Glucose (mg/dL) | Insulin  (μUI/mL) | HDL-C (mg/dL) | Triglyceride (mg/dL) | ASAT (mg/dL) | ALAT  (mg/dL) | Urea (mg/dL) |
| --- | --- | --- | --- | --- | --- | --- | --- | --- |
| 15 days | Control | 97.7 ± 9.9 | 10.0 ± 1.1 | 43.8 ± 1.2 | 77.0 ± 2.4 | 161.1 ± 16.8 | 70.8 ± 2.2 | 45.2 ± 9.4 |
|  | 15 ppm | 116.5 ± 6.3 | 10.5 ± 0.8 | 43.6 ± 1.5 | 79.2 ± 1.1 | 171.9 ± 12.9 | 69.0 ± 7.6 | 47.0 ± 7 |
|  | 32 ppm | 116.4 ± 8.0 | 11.7 ± 1.6 | 37.8 ± 0.6 | 83.2 ± 1.8 | 166.4 ± 11.4 | 71.6 ± 3.3 | 48.5 ± 5 |
| 1 month | Control | 94.4 ± 2.1 | 9.8 ± 1.3 | 42.1 ± 4.0 | 83.3 ± 4.7 | 166.9 ± 9.5 | 81.6 ± 3.2 | 47.0 ± 6 |
|  | 15 ppm | 120.7 ± 5.2▲ | 14.7 ± 1.1▲ | 37.8 ± 1.9▼ | 98.8 ± 1.9▲ | 168.7 ± 4.1 | 79.6 ± 12.1 | 48.4 ± 9.3 |
|  | 32 ppm | 117.1 ± 6.5▲ | 18.6 ± 1.9▲ | 37.6 ± 2.4▼ | 111.0 ± 2.4▲ | 176.0 ± 7.8 | 86.3 ± 14.0 | 50.1 ± 7.3 |
| 2 months | Control | 99.2 ± 6.3 | 10.1 ± 0.9 | 41.8 ± 3.8 | 79.0 ± 4.0 | 173.2 ± 10.5 | 81.9 ± 4.6 | 52.7 ± 7.4 |
|  | 15 ppm | 117.5 ± 7.3▲ | 16.8 ± 1.8▲ | 36.4 ± 1.5▼ | 98.2 ± 3.3▲ | 238.6 ± 8.3▲ | 105.2 ± 6.9▲ | 58.7 ± 4.5 |
|  | 32 ppm | 132.4 ± 8.0▲ | 21.6 ± 1.6▲ | 35.2 ± 1.2▼ | 101.4 ± 5.6▲ | 255.9 ± 6.7▲ | 115.1 ± 4.8▲ | 61.0 ± 6.9 |
| 3 months | Control | 101.7 ± 2.0 | 10.0 ± 1.4 | 40.4 ± 2.7 | 81.8 ± 2.6 | 184.1 ± 9.3 | 90.0 ± 2.5 | 55.3 ± 9.1 |
|  | 15 ppm | 124.5 ± 6.4▲ | 15.5 ± 2.7▲ | 34.6 ± 2.4▼ | 106.4 ± 2.7▲ | 193.2 ± 6.8▲ | 132.5 ± 10.1▲ | 72.0 ± 8.8▲ |
|  | 32 ppm | 140.4 ± 9.0▲ | 21.8 ± 1.9▲ | 35.2 ± 1.6▼ | 107.6 ± 2.8▲ | 317.9 ± 5.8▲ | 145.8 ± 9.2▲ | 78.0 ± 9.8▲ |
| 4 months | Control | 108.2 ± 2.0 | 10.1 ± 1.5 | 39.4 ± 2.3 | 82.0 ± 2.6 | 198.3 ± 5.8 | 97.3 ± 8.3 | 52.0 ± 5.6 |
|  | 15 ppm | 126.4 ± 8.5▲ | 19.4 ± 2.5▲ | 33.0 ± 0.9▼ | 127.6 ± 3.3▲ | 264.3 ± 7.1▲ | 139.4 ± 4.9▲ | 78.3 ± 9.2▲ |
|  | 32 ppm | 148.8 ± 4.8▲ | 20.3 ± 2.3▲ | 31.6 ± 1.0▼ | 133.8 ± 3.2▲ | 298.1 ± 9.3▲ | 150.8 ± 13.6▲ | 82.0 ± 7.7▲ |
| 5 months | Control | 104.5 ± 9.8 | 10.6 ± 1.3 | 38.2 ± 2.1 | 89.6 ± 2.8 | 204.2 ± 6.2 | 101.7 ± 12.4 | 54.1 ± 7.3 |
|  | 15 ppm | 137.2 ± 6.5▲ | 20.5 ± 2.8▲ | 31.8 ± 1.2▼ | 138.6 ± 5.2▲ | 310.8 ± 7.8▲ | 145.7 ± 9.8▲ | 82.6 ± 5.1▲ |
|  | 32 ppm | 159.2 ± 7.7▲ | 20.1 ± 2.7▲ | 28.6 ± 1.4▼ | 149.6 ± 8.5▲ | 352.9 ± 8.7▲ | 159.6 ± 6.4▲ | 85.0 ± 10.9▲ |

The results shown are the average of 5 different experiments ± SEM. (▲/▼) indicates a significant difference between the control group and Cd-exposed groups by a two-way repeated measures ANOVA followed by a Bonferroni test. A p-value of ≤ 0.05 was considered statistically significant. **HDL-C**, cholesterol transporter high-density lipoprotein; **ASAT**, Aspartate aminotransferase; **ALAT**, Alanine aminotransferase
